# Knowledge graph and its application in the study of neurological and mental disorders

**DOI:** 10.3389/fpsyt.2025.1452557

**Published:** 2025-03-18

**Authors:** Qizheng Wang, Fan Yang, Lijie Quan, Mengjie Fu, Zhongli Yang, Ju Wang

**Affiliations:** ^1^ School of Biomedical Engineering, Tianjin Medical University, Tianjin, China; ^2^ State Key Laboratory for Diagnosis and Treatment of Infectious Diseases, The First Affiliated Hospital, Zhejiang University School of Medicine, Hangzhou, China

**Keywords:** neurological disorders, mental disorders, knowledge graph, Alzheimer’s disease, Parkinson’s disease, depression

## Abstract

Neurological disorders (e.g., Alzheimer’s disease and Parkinson’s disease) and mental disorders (e.g., depression and anxiety), pose huge challenges to global public health. The pathogenesis of these diseases can usually be attributed to many factors, such as genetic, environmental and socioeconomic status, which make the diagnosis and treatment of the diseases difficult. As research on the diseases advances, so does the body of medical data. The accumulation of such data provides unique opportunities for the basic and clinical study of these diseases, but the vast and diverse nature of the data also make it difficult for physicians and researchers to precisely extract the information and utilize it in their work. A powerful tool to extract the necessary knowledge from large amounts of data is knowledge graph (KG). KG, as an organized form of information, has great potential for the study neurological and mental disorders when it is paired with big data and deep learning technologies. In this study, we reviewed the application of KGs in common neurological and mental disorders in recent years. We also discussed the current state of medical knowledge graphs, highlighting the obstacles and constraints that still need to be overcome.

## 1 Introduction

Neurological disorders and mental disorders are diseases that pose a large burden on worldwide health. Neurological disorders are a heterogeneous group of diseases that are characterized by the loss or dysfunction of the neurons in the central nervous system or peripheral nervous system (1), among which are epilepsy, common neurodegenerative disorders including Alzheimer’s disease (AD), Parkinson’s disease (PD) and multiple sclerosis, as well as cerebrovascular diseases such as stroke, migraine and other headache disorders. Neurodegenerative diseases can be classified according to primary clinical features (e.g., dementia, parkinsonism, or motor neuron disease), anatomic distribution of neurodegeneration (e.g., frontotemporal degenerations, extrapyramidal disorders, or spinocerebellar degenerations), or principal molecular abnormality (2). Mental disorders are usually characterized by a clinically significant disturbance in an individual’s cognition, emotional regulation, or behavior. The common types of mental disorders include depression, anxiety disorders, post-traumatic stress disorder (PTSD) and schizophrenia. Naturally intertwined and related to many common genetic, environmental and lifestyle factors, neurological disorders and mental disorders share some neurocognitive and pathophysiological mechanisms and are often suggested to be merged by some researchers (3–6).

The pathophysiology of neurological disorders and mental disorders is quite complicated and various factors may be involved, which greatly complicates their prognosis and clinical therapy. Over the years, a vast amount of medical data related to these diseases has been collected, which has become an invaluable resource for their basic and clinical study. However, the large volume, high variety and fast updating of the disease-related data also pose great challenge as disease research progresses, necessitating the development of novel strategies for big data processing, storage, and management. In such a situation, creation of new, scalable big data infrastructures and data analysis technique is urgently needed, which can help physicians to improve the efficiency of decision-making and outcome of patient care by extracting the information they need from medical data (7). Also, medical literature is frequently dispersed across multiple knowledge sources in various formats (e.g., websites compiling biomedical literature, databases of medical or clinical trial reports, electronic medical records), which causes difficulties to find pertinent data. Furthermore, it becomes more and more difficult to get trustworthy information to direct practice, research or clinical trials as the body of knowledge about diseases grows rapidly and some noises are inevitably included. Recently, knowledge graph (KG) has gained significant interest from both academia and industry as a type of structured knowledge. By visualizing complex concepts and creating links between them, KG can make data analysis and mining easier, and plays more and more important roles in biology, clinical treatment, data analysis, and other domains (8, 9).

### 1.1 What is knowledge graph

Knowledge graph, also known as a semantic network, is a directed labeled graph in which domain-specific meanings are represented by nodes and edges, with a node defining a real-world entity, such as objects, events, situations or concepts, and an edge capturing the relationship between two nodes. Such kind of graph-based representations of data are employed widely in computer science, especially in the field of artificial intelligence (AI). Indeed, choosing suitable representations to store information and derive new knowledge is central to AI and has drawn much interest in the past decades.

The concept foundation for KG development is related to the idea of semantic networks proposed in the 1960s. Semantic networks are graphical representations of knowledge based on meaningful relationships of text, structured as a network of words cognitively related to one another (10). Similar to KG, concepts are also represented by nodes and the connections between them are represented by edges in semantic networks, which allow the extraction of meaningful data by identifying emergent clusters of concepts rather than analyzing frequencies of isolated words (11). In the 1970s, the idea of ontology was first proposed for knowledge organization. The addition of ontology to semantic networks can encourage individuals to focus on the underlying relationships between entities rather than simply summarizing semantic data (12). Then, in 1989, Berners-Lee presented the idea of the World Wide Web, which adopts an interactive global information architecture that uses keywords to connect various sections of a document and enable interactive search (13). This system can recognize the connections between texts, enabling information retrieval beyond the constraints of merely following a path step-by-step. Additionally, the semantic web was proposed in 1998, which makes it possible for computers to process data from the web and analyze it (14). Consequently, search engines are now able to search content directly instead of merely on websites and greatly increases the effectiveness of searches. The concept of linked data was proposed in 2007, which enables us to construct typed linkages between data from various data sources via the Web. Resource description framework (RDF) is used to generate typed assertions that connect any two arbitrary objects in the world (15).

In order to improve search quality, Google launched the concept of the KG in 2012 based on the aforementioned technology (15). A KG is essentially a semantic network that reveals relationships between entities and allows for a formal description of real-world entities and their interrelationships. By building the “entity-relationship-entity” triangle, along with entities and associated attribute-value pairs that are connected by relationships to form a net-like knowledge structure, KG has some unique advantages in data representation and applications, and also makes it easier to conduct some interactive actions, such as inference, error correction and annotation. With the development of artificial intelligence technology, KG technology has played a vital role in fields such as intelligent search, intelligent Q&A, big data processing, and personalized recommendation (16). KG is also widely used in the medical field and is a hotspot of global artificial intelligence research.

In the medical field, KG has become one of the key factors for intelligent health care. By dealing with the uncertainty and ambiguity in medical data, KG is able to derive new knowledge and relationships, and has been used in a variety of intelligent medical applications. For example, KG is often used as the basis for medical knowledge retrieval, assisted diagnosis and treatment, as well as electronic medical records (17), which has greatly promoted the development of intelligent medical assistance (18). Actually, KG has become a fundamental component for AI aided medical systems such as the clinical decision support systems (CDSSs) for diagnosis and treatment, and the self-diagnosis utilities used to assist patients to monitor their health conditions (19). Recent years, KG has also been adopted in tasks like elucidation of molecular mechanisms of disease, pathway exploration, and drug discovery (20–23). With the development of KG and related technologies, KG will be crucial for future research in the biomedical field.

### 1.2 KG for the study of neurological and mental disorders

As we know, the knowledge required to capture the features of a certain disease or medical condition is not sufficiently represented in generic knowledge maps, which merely offer a general overview of medical or disease knowledge. In such a situation, disease-specific and targeted KG is necessary. Compared to generic KGs, the specific KGs are more accurate since they emphasize the coverage of entities related to a certain topic and concentrate on depth. However, creating medical KG for a specific disease takes a lot of time because of the complexity and volume of information in the medical area. There are two primary methods for creating KGs, i.e., a top-down approach and a bottom-up one. For the top-down approach, the knowledge base of already-existing and organized knowledge is constructed and the ontology and data schema for the KG are predefined before adding the entities. The majority of available medical KGs are created via the top-down approach. However, the KGs built this way have difficulties in providing a comprehensive picture of a specific component of the medical sector.

On the other hand, the bottom-up is a process of starting from a large amount of raw data, extracting entities, attributes and relationships through automated or semi-automated methods, and gradually constructing a KG. This approach is suitable for constructing open-domain KGs because it can handle massive data and extract information from it. Several different specialized medical KGs can even be connected to create a full-domain medical KG, which is beneficial for complex medical conditions in specialized domains (24). For example, we may create a KG of neurological and mental disorders based on the KGs of each common neurological disorder or mental disorder.

### 1.3 Medical knowledge graphs

**Figure 1:**
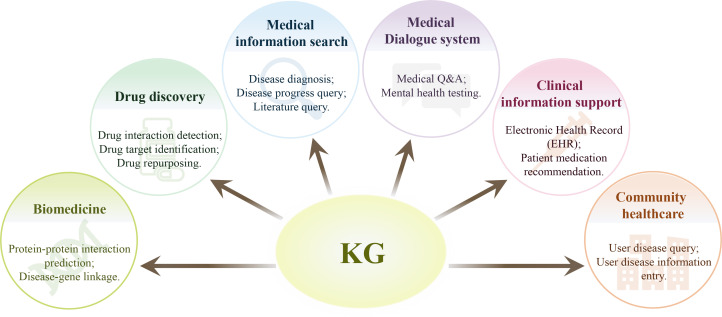
Application of knowledge graphs in medical filed. A medical knowledge graph may encompasses information from a large number of medical knowledge domains, and thus may play important roles in various domains of healthcare, such as biomedicine, drug discovery, medical information search, and other miscellaneous applications.

A medical KG is also made up of nodes and edges, with nodes representing medical entities like illnesses, symptoms, and therapies, and edges representing interactions or pertinent connections between nodes. Specific subject-related expertise is needed to build a medical KG, some of which can be acquired automatically from a range of data sources such as scientific publications, websites, textbooks, and real patient records, and some of which can be acquired manually (**Table 1**) (25). Regarding the healthcare domain, the non-interpretability feature of big language models, and their relatively poor performance on tasks involving contextual knowledge recall, correlation analysis, and decision-making, implicates that they cannot be used as a reliable artificial intelligence system. In such cases, medical KG provides another option that is able to improve in the efficacy of the healthcare industry at lower costs. A medical KG usually encompasses information from a large number of medical knowledge domains (26) (**Figure 1**).

**Table 1:** Some commonly used data sources for medical knowledge mapping.

Websites	Introduction	Specificities
PubMed	Free search system for biomedical literature developed by the National Center for Biotechnology Information (NCBI) of the U.S. National Library of Medicine (NLM)	PubMed is usually accompanied by a link to the full text, and the PubMed system features a toolbar that provides auxiliary search functions, a sidebar that provides other searches such as journal database search, subject term database search and feature literature search. The original text access service provides free titles and abstracts, which can be linked to the URL of the original text, and the search terms are automatically converted and matched, which is easy and fast to operate.
google scholar	A Google Web App for Searching Academic Articles for Free	The index includes most of the world’s published academic journals, providing an easy way to search a wide range of academic literature.
MeSH	The MeSH Medical Subject Headings database, known as Medical Subject Headings, was established by the U.S. National Library of Medicine.	MeSH is the authoritative subject headings list. It is a normalized, expandable and dynamic narrative thesaurus. It is used to include a vocabulary of biomedical terminology, which is used to describe the subject matter of each journal article in the medical literature database MEDLINE.
ClinicalTrials	It is currently the most important international registry for clinical trials.	Its registration and search for clinical trials are free of charge, and it is regarded as a model of open and international clinical trial registration.
WOSCC	The Web of Science Core Collection is the world’s leading citation database.	The Web of Science Core Collection database contains more than 12,000 of the world’s most authoritative, high-impact academic journals in the natural sciences, engineering, biomedical sciences, social sciences, arts and humanities, and other fields, dating back to as early as 1900.
DisGeNET	The DisGeNET database is a database of disease-related genes.	DisGeNET integrates data from multiple sources, including expert-managed repositories, GWAS catalogs, animal models, and the scientific literature. DisGeNET provides metrics and tools to help researchers explore and analyze the genetic basis of human disease. DisGeNET can be accessed through a web interface, Cytoscape App, RDF SPARQL endpoints, scripts, and R packages to access it.
SemMedDB	A PubMed-scale biomedical semantic prediction library.	A repository of semantic predictions (subject-predicate-object triples) extracted from the entire PubMed citation set. Serves as a knowledge resource to aid hypothesis generation and literature-based discovery in biomedicine, as well as clinical decision support.
LINCS	The Library of Integrated Network-Based Cellular Signatures	NIH’s database of perturbing cells by various means (e.g., knocking out, overexpressing certain genes, but mainly drug effects on cells) and then comparing cell expression profiles or other cellular processes before and after the cell perturbation.
IDG	International Data Group is the world’s largest information technology publishing, research, events and venture capital firm.	It has established a fast and comprehensive worldwide information network by adopting modern means of information processing and transmission, such as electronic mail, databases, telex and on-line services.
DrugBank	DrugBank is a free-to-use web-based database that provides bioinformatics and cheminformatics information, including detailed drug data and comprehensive information on drug targets and drug actions.	It combines detailed drug data with comprehensive drug target information, and the results are experimentally validated, authentic and reliable bioinformatics and cheminformatics databases.
UMLS	The Unified Medical Language System is a giant medical terminology system that has been under continuous development by the U.S. National Library of Medicine for over 20 years.	It contains about 2 million medical concepts and an unprecedented number of medical vocabularies, amounting to more than 5 million. UMLS has been applied in the areas of electronic medical records, health services, public health statistics, biomedical literature classification, and basic clinical and health services research.
KEGG	KEGG is a database resource for exploring high-level functions and utilities of the biological system (e.g., cell, organism and ecosystem) from molecular-level information.	The KEGG includes a series databases for biological information systems in the cell, the organism and the biosphere represented in terms of molecular interaction and reaction networks. It is a highly organized structure of data and knowledge aiming to model the real world. The KEGG is curated manually by capturing and organizing experimental knowledge reported in selected publications.

Medical KG can be used to visualize the subject-predicate-object triple content found in biomedical literature and databases, facilitating in-depth examination and the detection of connections between various diseases (27). In recent years, medical KG has been adopted in the study and clinic practice and has shown great potential for neurological and mental disorders, such as Alzheimer’s disease (AD) (28), Parkinson’s disease (PD) (29), attention deficit hyperactivity disorder (ADHD) (30) and depression (31).

## 2 Building medical KGs for neurological and mental disorders

In recent years, a number of knowledge graphs for neurological and mental disorders have been built by different groups (**Table 2**). In the following sections, we will provide an overview of these valuable resources.

**Table 2:** Knowledge graphs constructed for neurological and mental disorders.

Authors	Name of KG	Data Source	Objective
Goodwin et al. (32)	QMKG	95,703 de-identified EMRs from multiple hospitals during 2007	To provide a method for automatically constructing a clinically relevant concept map based on belief states.
Cheng et al. (33)	SMKG	Medical thesaurus, ICD-10 coding, and other medical terms as entities in the entity resource database	Assisting in the construction of a smart question and answer and medical assisted decision making system for stroke.
Wytze et al. (34)	none	Euretos Knowledge Platform	Using predictive information to determine disease trajectories.
Papadakis et al. (35)	ADHD-KG	MeSH, PubMed, Clinical Trials, Side Effect Resource, DrugBank	To provide valuable assistance to researchers and clinicians in the study, training, diagnosis, and treatment of ADHD.
Kaur et al. (36)	none	PubMed, healthboards.com, psychforums.com, reddit	To improve knowledge mobilization, communication, and care for individuals with ADHD and ASD.
Costello et al. (24)	none	Included individuals with lived experiences who were part of the family advisory board or were recruited through advertisements for the project and community support groups focused on NDDs, the Inform Alberta	To help health professionals, support groups, and families share, combine, and access the resources of NDDs.
Huang et al. (26)	DepressionKG	PubMed, Clinical Trials, Medical Guidelines, DrugBank, Wikipedia, DrugBook, SIDER, UMLS	To gain a more comprehensive understanding on depression.
Liu et al. (37)	MiKG4MD	PubMed, Google Scholar	Identifying, exploring, and predicting the relationship between the gut microbiota and mental disorders.
Sun et al. (38)	MMiKG	PubMed, Springer, Google Scholar	As a guideline for clinical and biological experiments, it opens up new avenues for therapeutic strategies for psychiatric disorders.
Nian et al. (39)	none	PubMed, SemMedDB	Predict reliable new relationships between AD and other entities.
Scott et al. (28)	none	PubMed, UMLS	Combining machine reading and KG can augment human expertise in causal feature selection.
Soman et al. (29)	SPOKE	EHR data of patients who visited UCSF between 2010 and 2020	Can provide early prediction of Parkinson’s disease in a clinically interpretable manner.
Yang et al. (40)	KGAP	LINCS, NCBI Gene Expression Omnibus (GEO), IDG	For efficiently searching and summarizing evidence pathways based on disease queries to identify, score and rank relevant genes as drug target hypotheses.
Li et al. (41)	none	Web of Science Core Collection (WOSCC), Social Science Citation Index (SSCI), Current Chemical Reactions (CCR-EXPANDED), Index Chemicus (IC)	Explore the current state of Parkinson’s disease research, research hotspots, and cutting-edge areas.
Pu et al. (42)	None	PubMed, bibliography of Alzheimer’s Disease literature, Neuropsychological Integrative Ontology (NIO), Pubtator Central	A literature-based discovery (LBD) model for link prediction and graph embedding learning for Alzheimer’s Disease.
Fu et al. (43)	MDepressionKG	KEGG, MENDA, Microbe-Disease Knowledge Graph (MDKG)	A knowledge graph linking metabolism entities of human and their microbes to depression disorder.
Fu et al. (44)	Food4healthKG	FoodData Central dataset (FDC), FoodOn, Chinese Food Ontology, KEGG, NCBI taxonomy, MENDA, and SNOMED CT	A comprehensive knowledge graph focusing on food, gut microbiota, and mental diseases.

### 2.1 Data collection

As health information systems often accept data in both organized and unstructured formats, extracting and mining the irregular information from the specialized sources is always a challenge. There are some valuable and popular sources of medical knowledge data. Research articles deposited in PubMed or Web of Science are widely used in some studies. Scott et al. derived the knowledge of AD by analyzing the articles deposited in the PubMed database (28). Nian et al. collected biomedical entities and their interactions from the literature in PubMed, including the connections between chemicals, medications, and dietary supplements and AD (39). Pu et al. constructed an AD corpus of over 16,000 articles published between 1977-2021, which was automatically annotated with concepts and relations covering eleven AD-specific semantic entity types (42). The articles were collected by an expert in AD study, who conducted a biweekly literature search on the Web of Science using keywords related to AD. From the search results, publications were chosen based on the knowledge of the expert. To study postural control in PD patients, Li et al. indexed the literature in the Web of Science Core Collection (WOSCC) by keywords postural balance, postural control, and Parkinson’s disease, and retrieved 1347 original papers comprising 64631 references, based on which a Citespce KG for PD was built (41). ADHD-KG, a specific KG for ADHD, was built by integrating data from a number of medical sources, including the DrugBank, Clinical Trials, Side Effect Resource, PubMed, and Medical Subject Headings (MeSH) (35). Huang et al. (26) developed a knowledge-based actual patient data generator, APDG (45), and concentrated on knowledge resources related to the use of antidepressants. They employed data sources such as PubMed, Clinical Trials, Medical Guidelines, DrugBank, Wikipedia Antidepressants, SIDERS, and UMLS.

Professional websites are also important data sources for medical KGs. Costello et al. (24) built KG for neurodevelopmental disorders (NDD) based on text corpus from two sources. One was the authors’ compilation of the NDD caregiver subset consisting of community members and organizations connected to NDD, and the other was the Inform Alberta website (http://www.informalberta.ca). The author combined a list of pages from these two sources and other relevant web pages using the Python Scrapy package. To create a stroke KG, Cheng et al. gathered stroke-related medical data from two medical websites, Dingxiangyuan and Xunyiwenyao.com (33). In order to prevent data omission, they also gathered pertinent data from the Chinese Symptom Database of the East China University of Science and Technology (33) and the Baidu Encyclopedia. A KG known as MMiKG was created by Sun et al. (38) for microbe-gut-brain axis and its intricate correlation with mental disease. They examined PubMed, Springer, Google Scholar, and other literature search databases using the terms depression, schizophrenia, anxiety, autism, and bipolar illness, and collected a total of 907 pertinent publications on the gut-brain axis and mental disorders (38).

Electronic health records (EHRs) are another frequently used data source. Electronic patient medical records gathered at the University of California, San Francisco (UCSF) between 2010 and 2020 were used by Soman et al. (29) to develop a KG for PD. To verify the accuracy of the data, patients were divided into two groups based on the PD diagnostic codes found in the EHR diagnostic table. Additionally, instances of both genetically and drug-induced PD were removed.

### 2.2 Entity extraction & relationship identification

#### 2.2.1 Entity extraction using databases and documentation tools

Creating KGs for content in unstructured texts involves extracting concepts, events, and relationships. To increase accuracy, entity identification should take into account both new and previously stated concepts, events, and illnesses in the vocabulary. Because most medical entities have synonyms and practitioners may use different names to refer to the same item, entity creation is an important step, which requires mapping each entity to a uniform entity set (46).

Unified Medical Language System (UMLS) is a set of files and softwares that brings together many health and biomedical vocabularies and standards to enable interoperability between computer systems (47). With more than 3 million concepts based on nearly 200 sources, it integrates and distributes key terminology, classification and coding standards, and associated resources to promote the development of more effective and interoperable biomedical information systems and services, including electronic health records. SNOMED-CT (Systematized Nomenclature of Medicine-Clinical Terms) and MeSH (Medical Subject Headings) are also commonly used resources. To extract UMLS entities from text, Costello et al. (24) employed the UMLS Entity Linker in the open-source framework SciSpaCy. In building a KG for attention deficit hyperactivity disorder (ADHD), Papadakis et al. (35) recognized the meaningful terms and associated them with MeSH concepts through ScispaCy, which had the ability to parse medical abbreviations and provided entities directly linked to MeSH concepts. As MeSH concepts are systematically linked to different resources, their approach is able to capture conceptual associations between entities and can enhance and accelerate information retrieval.

Using SemMedDB, Nian, et al. collected biomedical annotations and extracted their relations and filtered 1,672,110 AD-related semantic triples, which were used to train with multiple KG completion algorithms to predict candidates that might be helpful for AD treatment or prevention (39). Scott et al. (28) introduced a novel KG application enabling causal feature selection by combining computable literature-derived knowledge with biomedical ontologies. They retrieved computable knowledge from a literature corpus using machine reading systems SemRep (48, 49) and INDRA (50) to extract triples, and mapped the output to target terminologies and combined with ontology-grounded resources. Then, the model was used to estimate the total causal effect of depression on the risk of developing AD from observational data. To build a KG for depression, Huang et al. (26) collected information related to depression from various resources, e.g., publications deposited in PubMed, clinical trials (https://clinicaltrials.gov/) and drug and target information in DrugBank (https://go.drugbank.com/). Then, the collected data were integrated based on identifications such as PMID, MeSH or medical terminologies.

Sun et al. developed a knowledge graph MMiKG for the microbiota-gut-brain axis (38). As knowledge related to the regulation of the host central nervous system by gut microbiota is fragmented and usually included in disorganized or semi-structured unrestricted texts. They collected the information by scrutinizing literature and merged various associated resources and deducing prospective connections between gut microbiota and the central nervous system. They gathered 1,257 triads through human data integration and assessment (38). To explore literature-based knowledge for AD and predict new knowledge, Pu et al. built a KG for the disease (42). They collected an AD-specific corpus from over 16,000 publications and two biomedical knowledge resources, Neuropsychological Integrated Ontology (NIO) (51) and Pubtator Central (52), to construct an AD KG consisting of about 11,000 entities and 394,000 relations, which could be used to infer new knowledge with graph embedding-based link prediction methods.

Another popular source of information is corpus collection from community-based forums. To explore the relationship between depression and consumption of cannabis, Roy and colleagues gathered 11,000 tweets using the Twitter API (53). The information was analyzed by natural language processing techniques to generate a targeted social media corpus involving personal use of cannabis with the intent to derive its potential mental health benefit. Then, the data was combined with domain knowledge from the Drug Abuse Ontology (54) and Diagnostic and Statistical Manual of Mental Disorders (DMS-5). Their experiments showed the method could significantly extract cannabis-depression relationships.

#### 2.2.2 Relationship identification using AI tools

Even if the intricate relationships between diseases are beyond the current capabilities of natural language processing techniques, these techniques can nevertheless be useful in the construction of KGs, and large language models can often make relationship extraction easier and more effective.

Roy et al. proposed a framework based on supervised contrastive learning and GPT-3 to extract entities and their associations, which was adopted to explore the relationship between depression and consumption of cannabis in a targeted social media corpus and built a domain-specific drug abuse ontology (DAO) (53). With the new tool, they retrieved a corpus from community websites with improved performance and high-quality annotations, which made it possible to comprehend the connection between marijuana and sadness. Natural language processing methods were employed for semantic annotation by Huang et al. (26), in which Xerox’s NLP tool XMedlan was used to semantically annotate medical texts using medical terminology like SNOMED CT.

### 2.3 Entity completion and knowledge fusion

In building KG, knowledge fusion is essential when there are multiple data sources. By combining data in distinct formats into a single entity, knowledge fusion can facilitate the unification of data gathered from different sources.

Knowledge fusion for the stroke MKG was handled from two angles by Cheng et al. (33), i.e., entity attribute alignment and entity linking. The process of aligning entities from knowledge bases of disparate data sources into unique identifiers for real-world entities is known as entity alignment. The aligned entities are then connected to the KG for stoke. The diagnosis of depression usually depends on subjective assessments and clinical interviews, which often causes potential biases and inaccuracies. Yang et al. proposed a new framework using multimodal data for depression diagnosis (55). To integrate the diverse data modalities like textual, imaging, and audio information, and tackle the challenges of data heterogeneity and high dimensionality, representation learning was adopted to autonomously discover meaningful patterns and features from the data, and knowledge transfer was adopted to transfer knowledge from related domains. Results indicated the new approach significantly improved the diagnosis of depression.

### 2.4 Data processing

Data that is kept in a set format and organization is referred to as structured data. Database tables, fields, and data types can be used to describe the relationships between data items, which are characteristics of structured data. Structured data also has a well-defined organizational structure. On the other hand, unstructured data refers to data that do not have a fixed format or structure, which cannot be easily stored and processed using traditional relational databases. Since unstructured data is characterized by a free and irregular format, which usually requires special processing to extract useful information.

In building the KG for stroke, a distributed crawler was utilized by Cheng et al. to autonomously collect medical data (33). To do this, lightweight JSON files were created from structured data gathered from three databases, i.e., vertical medical websites, crowdsourcing websites and public knowledge base. Additionally, the data was checked to eliminate any characters that were missing, jumbled, or prohibited. In building their KG for ADHD, Papadakis et al. converted the data in XML or CSV formats into RDF (Resource Description Framework) (35). In order to overcome structural discrepancies, Murali et al. employed representation learning to accurately represent multimodal data with structured knowledge and language from numerous sources (56, 57). Multimodal learning was employed for improved inference, classification, and prediction, depending on the nature of the data representation (46). Costello et al. (24) used the Python BoilerPy3 package to extract the HTML text from each page and then cleaned it up by deleting sample text. The document corpus utilized to construct the KG-based NDD repository was made up of the clean HTML content that was extracted from the pages.

### 2.5 Information linking

Missing linkages in the KGs for diseases are often important for their diagnosis and treatment. For example, diagnostic predictions require the extraction of new and meaningful relationships utilizing a variety of techniques from existing entities and relationships.

To link the entities, Costello et al. assigned a weight to each entity based on how frequently the entity occurs in the data collected for NDD (24). These weights were known as entity-related weights, and Latent Dirichlet Allocation (LDA) was used to determine the topics for the data source, and the hierarchical structure of subjects was employed in topic modeling to create an NDD knowledge graph by extracting related topics from the corpus. The strength of the linkages between themes and entities was determined using the LDA algorithm.

Based on the article title and abstract, Pu et al. established a relationship between two entities through co-occurrence (42). They claimed that rather than using causality as a relationship, they opted to use co-occurring associations because the first attempts at extracting semantic triples using SemRep produced errors and had inadequate coverage of concepts and relationships related to AD. In order to address this issue, Scott et al. conducted a study on the causal link between depression and AD (28). Refining ontology-based knowledge in a knowledge graph helped to partially address the shortcomings of structured knowledge generated from books. Additionally, they demonstrated how semantic reasoning could improve causal inference while also offering an effective means of representing knowledge and automating reasoning to produce mechanistic hypotheses about biomolecular processes of confounding factors. A reweighting process was employed by Scott et al. to condense the knowledge graph into biologically significant things that were helpful for AD research.

RDF supports semantic representation of data, allowing the expression of complex relationships through predicates, and can be nested to use, forming complex data structures. RDF data can be represented in a variety of formats, such as RDF/XML, Turtle, JSON-LD and so on. Two methods of data linking were proposed by Papadakis et al. (35), i.e., indirect informational linking is defined as when various data sources contain resources that reference common medical concepts, whereas direct informational linking is defined as when a data source explicitly references a resource defined in a class of external datasets. Whatever the kind of linking, though, custom RDF statements that associate related resources express the relationship between the data sources.

### 2.6 Knowledge graph embedding

A common data representation method for KG is knowledge graph embedding, which transforms graphs into low-dimensional vector formats. In order to compute entity similarity, graph embedding preserves semantic information between entities in KG and learns the entities and their relationships in the form of distributed embeddings (58). Clinical applications have benefited greatly from the use of knowledge graph embeddings. One of the functions of knowledge graph embeddings, which are still in their infancy in the clinical domain, is to perform ternary characterization using convolutional neural networks or to capture the underlying semantics of ternary groups for semantic matching. Link prediction can also be treated as a binary classification problem to construct a graph embedding model, from which new knowledge can be inferred through graph embedding-based link prediction methods (42). By comparing various link prediction techniques within the framework of short- and long-term knowledge evolution in AD, the effects of restricting the predictive evaluation of LBD models can be assessed. Then, knowledge graph embedding can be used to predict candidates that might be helpful for AD prevention or treatment (39).

### 2.7 Visualization of knowledge graphs

Knowledge graph representation can help understand the entities and their relationships intuitively, and also make it easier to find hidden information and unidentified associations (38). Based on their visual layout and encoding, knowledge graph visual representations fall into several categories, i.e., space filling, heat maps, node-link diagrams, adjacency matrices, and certain non-primary visual representations like Euler diagrams and indented lists, among others. In order to execute semantic queries and link prediction, Sun et al. (38) stored the triples of various connection kinds in CSV files, loaded them into the Neo4j database, and displayed them using GraphXR. Citespace V software was used by Li et al. (41) to visualize and analyze the content of literature in terms of number of publications published each year, partnerships between institutions and countries, partnerships between authors, cited journals, and co-cited journals, to learn about the state of the research on postural control in people with PD.

### 2.8 Knowledge discovery

Knowledge mining, including knowledge content mining and knowledge structure mining, is the process of extracting new knowledge from already-existing entities and their connections. Rule mining is the primary function of knowledge structure mining, whereas entity linking (e.g., synonym discovery, disambiguation) belong to content mining.

**Figure 2:**
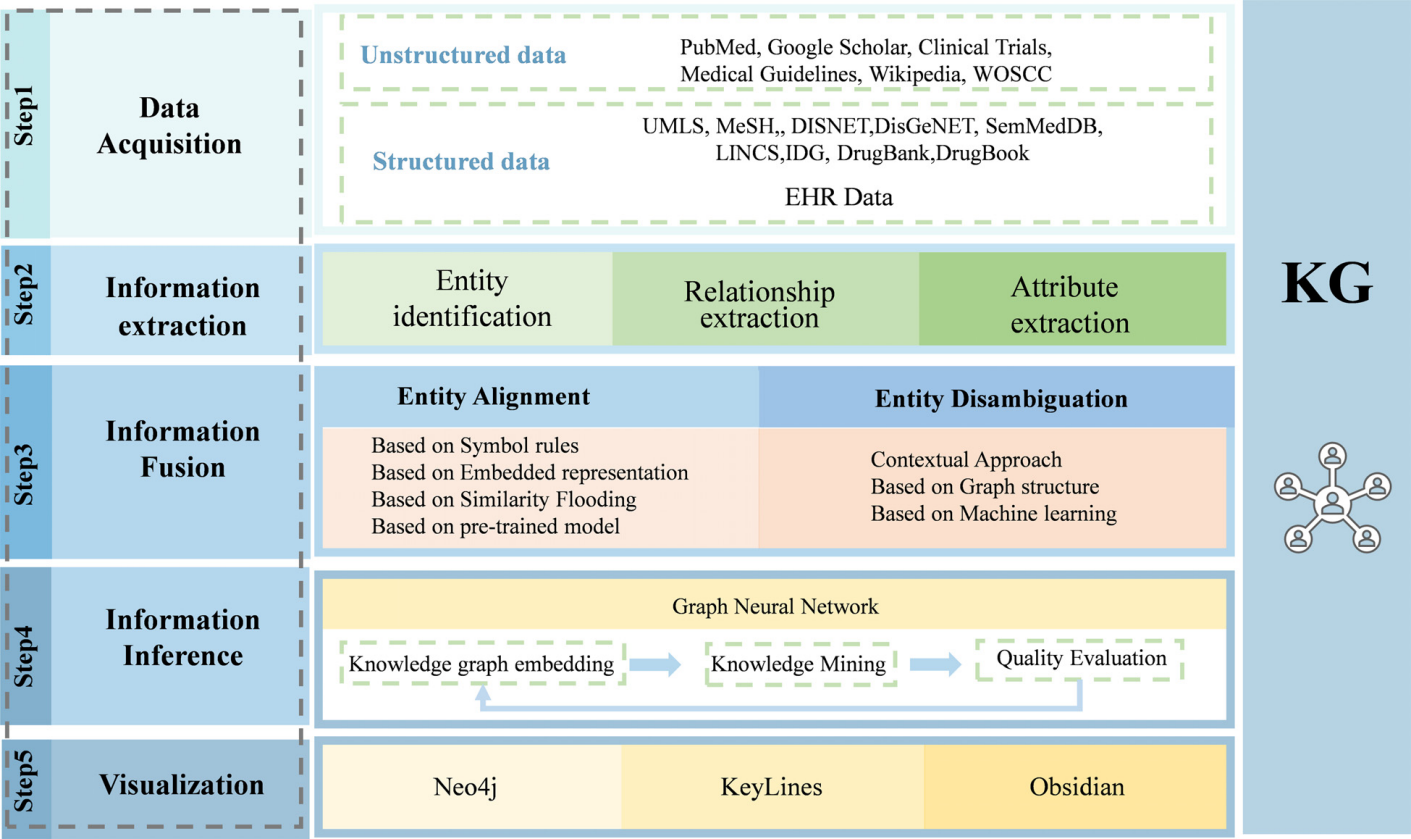
The procedure to build a medical knowledge graph. Building a knowledge graph involves several steps, including data acquisition, information extraction, information fusion, information inference, and knowledge visualization. By this procedure, data from different resources are integrated and represented as knowledge graph.

In their KG for microbiota–mental diseases, Sun et al. used Graph Data Science (GDS) and GraphXR embedded graphical algorithms to measure the relationship of edges, as well as to analyze each entity node of microbiota, intermediates, and diseases (38). By focusing on the representation of “facilitation”, “inhibition” and “inhibition”, they were able to analyze the data more thoroughly and find new paths of microbiota–mental diseases interactions. The above steps and techniques related to the construction of the KG are shown in **Figure 2**.

## 3 Application of KG in neurological and mental disorders

### 3.1 Outcomes and impacts of KG in neurological and mental disorders

The application of KGs in neurological and mental disorders has increased rapidly in recent years. Scott et al. used Dijkstra’s shortest path search to select causal features between depression and AD from the KG based on biomedical literature and ontologies (28). Machine reading concepts and inferred edges were utilized to increase the range of variables, or to make machine reading more comprehensive. Next, KGs based on ontologies were queried. To increase the accuracy of knowledge acquired from literature, filter the material using ontology knowledge. Furthermore, to offer speculative explanations for the causal association between the detected variables and depression and AD, visual inference pathways are developed. In order to infer implicit relationships in the KG of AD, Pu et al. (42) used graph embedding-based link prediction on 20 time-sliced datasets. They discovered that the link prediction task became increasingly challenging over time as the feature network density fell with increasing node count. In other words, outcomes from long-term prediction contexts differed significantly from those from short-term prediction contexts. This underscored the importance of carefully considering LBD-based techniques for AD evaluation.

The KG-based NDD repository created by Costello et al. was the first resource that combined reliable web resources from different areas into a unified database (24). The uniqueness of the database was its domain specificity, which included various information extraction techniques and incorporated patient-centered data from various sources (24). They developed a knowledge base for NDD including contributions from NDD-affected patients and caregivers, as well as medical experts with expertise in NDD. As a result, they created an automatic labeling pipeline for NDD-related web resources and a KG with a wider variety of NDD information. This work showed how artificial intelligence (AI)-based methods (such as KGs for information representation and natural language processing) could improve knowledge mobilization, extraction and be applied to various medical fields. By annotating web pages using a range of natural language processing methods and storing them in a structured KG, users could query the system by using text terms easily.

ADHDKG was created by Papadakis et al. (35) to streamline the retrieval of knowledge regarding ADHD and establish the framework for efficient medical question-and-answer sessions. In order to shift from laborious manual reviews of the medical literature to automated semantic searches of coded knowledge, the authors integrated knowledge regarding ADHD into a single resource and be searched by the powerful SPARQL queries. The efficiency benefits depended on ADHDKG’s capacity to comprehend the most recent developments related to ADHD research deposited in literature, which made ADHDKG a useful tool for research on ADHD.

To investigate the connections between chemicals, medications, dietary supplements and AD, Nian et al. built a KG based on information retrieved from literature (39). Their goal was to identify potential interventions to stop or slow the course of the neurodegenerative diseases. By using the knowledge graph-based techniques for AD medication repurposing, they were successful in finding data to support the potential efficacy of drugs for AD treatment, including prednisolone, tacrolimus, vasiclovir, and sebaceous steroids, all of which have been shown to be beneficial in the management of AD (59). Additionally, this approach could find proof for drugs that could prevent AD, such as oxytocin, betaine, and amphotericin (60, 61).

Based on the knowledge of the microbiota-gut-brain axis, Sun et al. constructed MMiKG, a knowledge graph-based platform for path mining of microbiota-mental disease interactions (38). It provided information on the connections between the gut microbiota and mental diseases, and supplies tools to mine the detailed relationship between the entities. Simultaneously, MMiKG could serve as a roadmap for biological and clinical research, which may be useful for exploring novel approaches to treating mental illnesses.

In order to reflect the current research status, research hotspots, and future development trends in the field of postural control in Parkinson’s disease patients in a more intuitive, effective, and scientific way, Li et al. (41) visualized and analyzed the literature about the field of postural control in PD patients.

### 3.2 KGs for depression

Major depressive disorder (MDD) is a common mental disorder that affects about 6% population globally (62, 63). With a lifetime incidence of 16.6%, MDD is among the most burdensome disease worldwide (64–67). In the United States alone, depression causes about 400 million disability days per year and an annual economic burden of $210 billion (68). It is estimated that MDD will become the major cause of Years Lost to Disability (YLD) in 2030 (69). MDD can affect many aspects of the patients, and it is also the leading cause of suicide (70, 71). The symptoms of MDD are complicated, including anxiety, cognitive impairment, suicidal tendencies, as well as emotional, somatic and functional impairments (72). The current available options for the prevention, diagnosis or treatment of depression have limitations, and it is still a huge challenge to develop more effective therapeutic approaches for MDD.

In recent years, the application of KGs to MDD study has received much attention. Huang et al. constructed KGs to describe depression based on data collected from a variety of major public knowledge sources, such as PubMed, Medical Guidelines, DrugBank and Unified Medical Language System (UMLS) (26). Li et al. proposed to use UMLS-based semantic prediction programs SemRep and SemMedDB to construct a KG for describing depression in a bottom-up way (73). Depression and its association with metabolism is also an interesting topic (43). Fu et al. developed MDepressionKG, a KG that integrates metabolic-related data involved in human microbial metabolism network, human diseases, as well as microbes, to build semantic-based rational reasoning and probable relations between depression and comorbid diseases. Yu et al. presented a hierarchical structure of depression knowledge network based on a systematic analysis of depression (74). By using softwares including Citespace, Ucinet, and Pajek, they employed the bibliometric methodology to analyze 5,000 research articles concerning depression. The constructed depression knowledge network could be helpful for understanding the hot spots, evolutionary trends, and future related research directions of the disease. There are also studies that combine KGs of depression and tools like machine learning to improve the detection of depression (31).

While these KGs can serve as useful resources to infer the knowledge of depression, they are usually based on a few major public databases or focus on certain aspects of the disease. To develop more personalized diagnostic strategies and targeted treatments, a deep understanding of the biological mechanisms of depression and the ability to dissect the relationship between molecular and genetic factors and their phenotypic consequences is necessary.

**Figure 3:**
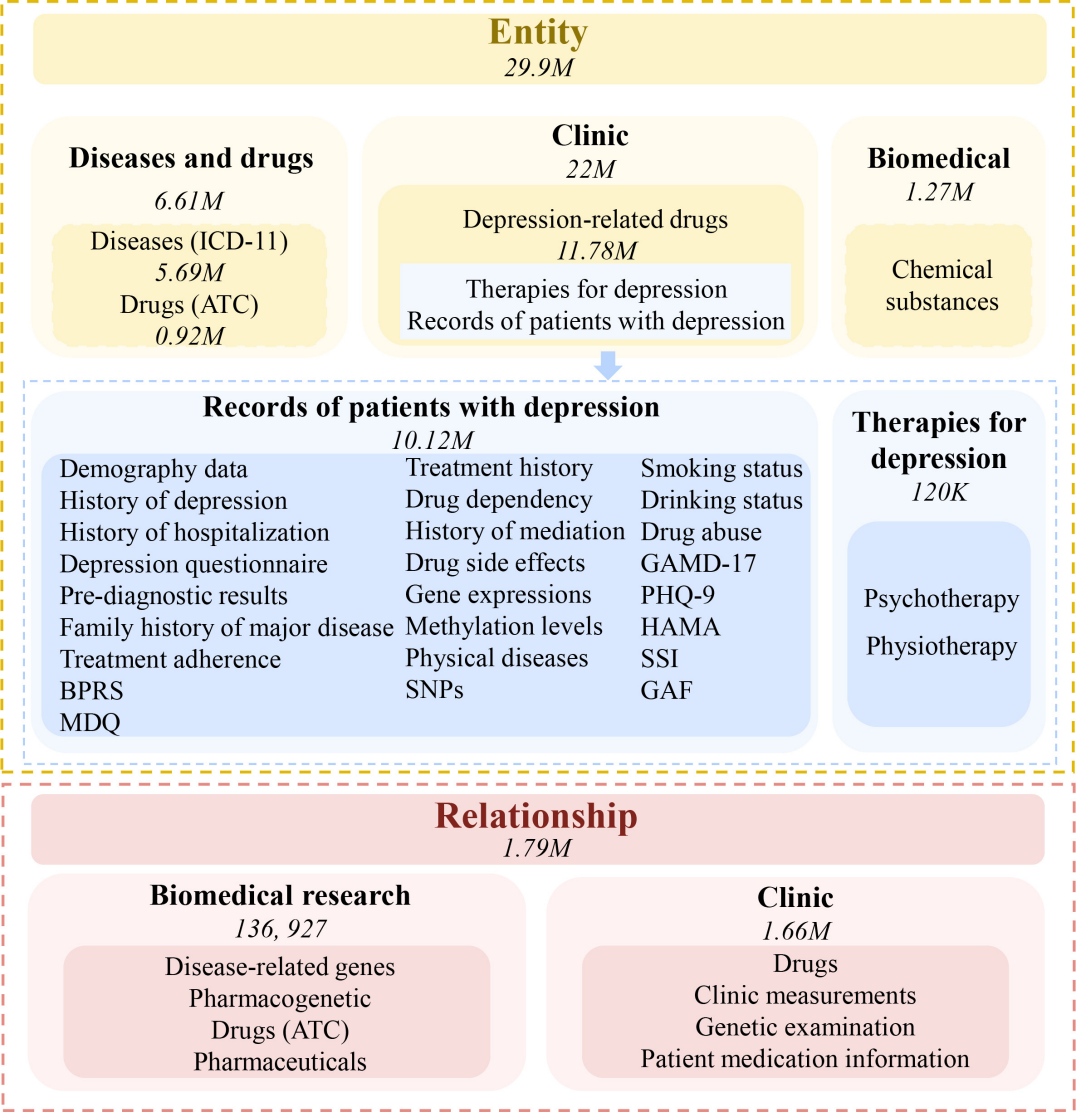
Knowledge graph for major depressive disorder. By collecting data related to the disease, a large entity-relationship repository including about 30 million entities and more than 1.79 million relationships has been built. These entities and relationships cover almost all aspects of the disease. The numbers in the figure represent the entities or relationships collected.

By using a variety of information gathering techniques, we created a new depression KG that integrates both basic medical information and clinical information. First, we conducted a comprehensive collection of depression-related information from a variety of public resources, such as DisGenNET (https://www.disgenet.org/), MalaCards (https://www.malacards.org/), KEGG (https://www.genome.jp/kegg/), Reactome (https://reactome.org/). Then, we collected the clinic data from a cohort of patients diagnosed with MDD. With this information, we have built a large entity-relationship repository, including about 30 million entities and more than 1.79 million relationships. These entities and relationships cover almost all aspects of depression, making it a quite comprehensive knowledge map of the disease (**Figure 3**). Next, we used ogdg-molpcba (OGB from MoleculeNet) and clinical data of patients collected by us to fine-tune the model for depression by two AI tools, Graph-ToolFormer (75) as well as GraphGPS (76). Based on this disease-centric MDD KG, users can quickly identify knowledge links spanning numerous knowledge resources and investigate the connections between different sources of information related to depression. We are developing models and algorithms that can help to diagnose depression or develop potential treatment plans. Hopefully, we can precisely diagnose subjects with depression and determine effective therapies for the new patients, including whether antidepressant medicines are acceptable and whether psychotherapy or physical therapy is needed, by comparing their genetic and clinic information with that included in the MDD KG.

## 4 Discussion

The “entity-relationship-entity” ternary structure used by KGs to represent knowledge is easy for computers to read and process, which can also help the user to gain insight into the underlying meaning of data and resolve the ambiguity and confusion while generating new information and connections. KGs can also depict more intricate knowledge systems at the same time, including a real-time updates of data and knowledge status.

With the dramatic increase in the speed of data generation along with the huge volumes of data accumulated from dispersed sources, the medical domain has been overwhelmed by big data, which provides a foundation for further scientific breakthroughs in both research and clinic practice in healthcare. However, such big data also poses challenges to mine knowledge and derive insights for developing more effective diagnoses and treatments for diseases. By representing knowledge in structured and dynamic ways, KGs can effectively represent and process complex information within a machine-readable context, and play a central role in representing information for AI systems. With KGs, users can go deeper into the relationship chain, which supports complicated inquiries in the field of medicine. These advantages make KGs an essential part of biomedicine and health informatics, especially in the field of neurological and mental disorders.

Large language models become more intelligent as AI develops, and the emergence of new AI tools opens up avenues for their application in the medical field. However, because large language models have a black-box mechanism, there are flaws in the quality of their output (77), and the veracity and accuracy of their sources cannot be guaranteed, which may limit the application of AI in healthcare. In the medical sector, it is crucial to make sure that the results can be tracked down and that the data sources are reliable and trustworthy. In this situation, knowledge graphs’ white-box mechanism can essentially explain the results generated, and the knowledge graphs’ traceability and authentic data sources can be used to construct better medical AI system (78). This can perfectly complement the most recent advancements in AI and its applications in healthcare and open up new avenues for the field’s growth.

Although KGs have advantages over big language models for their precise knowledge sources, they also have problems like information losses due to errors in unstructured data sources. In the medical industry, there is a significant emphasis on data source accuracy, especially for KGs related to medicine. The validity of the KGs will be directly impacted by inaccurate, lacking, or out-of-date information in the data source, But it is still difficult for researchers to determine which data sources are reliable and trustworthy. Thus, it often takes a lot of time and resources, including specialist expertise, technological tools, and computational resources, to create and maintain KGs. In the meanwhile, while KGs can combine data from multiple sources, they can help to address the issue like data silos. With the development and adoption of data sharing initiatives (e.g., FAIRsharing (79)), databases and repositories with shared infrastructure that enables data interoperability is essential for advancing research utilizing medical KGs. However, challenges such as privacy protection, ethical considerations, organizational culture, and differences in legal frameworks present significant barriers (80), and privacy protection and security measures are needed in industries like healthcare due to the sensitive information included in KGs.

## 5 Future and development

In future, increasing usage of modern KGs for neurological and mental disorders will not only significantly advance our understanding of these complex diseases, but also be advantageous for future clinical applications such as patient stratification or therapy selection. However, the development and application of KGs for neurological and mental disorders are still in the early stages and face many challenges.

While existing knowledge graphs for mental and neurological disorders have provided insights into understanding the diseases and shown potential in diagnosis and treatment, most of them only focus on individual disorders and have limited coverage. How to extend knowledge graphs to new domains of knowledge or different diseases poses a great challenge. This is particularly true in the mental health sector due to the complexity of the diseases and related data. For example, there are hundreds of mental disorders, and each may share common symptoms and molecular mechanisms with one or more other diseases. At the same time, while new tools like UMLS provide great convenience processing medical information, a large fraction of medical records are in languages other than English, how to integrate numerous heterogeneous data collected from multiple sources in various languages pose huge challenges (81). In such a situation, methods that can handle medical information with specific grammatical and semantic features according to the professional characteristics and medical background of different languages and cultures are highly desired. Of course, building KGs for neurological and mental disorders are only one aspect of creating a broad medical KG; in the future, knowledge graphs from various medical disciplines will work together to form generalized medical KGs. Therefore, figuring out how to combine and connect these KGs is also a crucial component of their development and application.

Currently, extracting entities and relations of medical knowledge graphs requires expert review and labeling to ensure accuracy of the data, which demands considerable time and resources. Manual processing, however, face huge challenges when dealing with large and wide datasets. Knowledge graphs that can collect information about entities and relationships automatically and update themselves are expected. To automating the extraction of knowledge extraction and regular updating of the database, advanced technology such as deep learning, and evolutionary algorithms, may be promising to overcome these limitations. Utilizing deep learning approaches to enhance knowledge graph representation and inference accuracy is a recent trend in the medical industry. Since data related to diseases like the neurological and mental disorders are dynamic and scalable, future research should focus on filling in the gaps in medical knowledge graph complementation. Some new techniques, such as Link Prediction (LP), can be used to predict missing information among entities already in a KG, and is a promising approach to address KG incompleteness (82). By a combination of dimensionality reduction techniques and graph neural networks, LP can reduce the dimension of high-dimensional feature spaces in network datasets while preserving relevant information (83). Such an approach can improve the quality and efficiency of biomedical KGs, and can be used to solve problems like drug repositioning (21). Especially, the synergies of KGs and large language models may facilitate a more in-depth integration of multimodal data and make KGs more accurate and easier to validate (9).

Future research should also make medical knowledge graphs more interpretable, which will boost outcomes and accuracy in technology-assisted medical decision-making (25). In biomedical knowledge graphs, rule-based reasoning based on knowledge graph relationship rules is usually used (84). Since the cost of manually obtaining these complex rules is relatively high, machine learning and deep learning algorithms can be used to mine potential rules of KGs. However, we still need to develop algorithms with lower complexity and fewer computational resource requirements (85). At the same time, new techniques related to KG should also be integrated into the development of medical knowledge graphs. For example, EMPWR, a comprehensive KG development and lifecycle support platform using a broad variety of techniques from symbolic and modern data-driven systems, has shown potential for creating and maintaining KGs for the pharmaceuticals domain (86).

Also, how to apply knowledge graphs to solve problems in research related to neurological or mental disorders is of great importance. While available KGs for neurological and mental disorders are mainly used for knowledge management, information retrieval and query, and unknown relations prediction (9), some novel applications are also under development. For example, Food4healthKG, a comprehensive KG focusing on food, gut microbiota and mental diseases, can be used for knowledge query and design of proper diet patterns for patients with mental disorders (44). KGs are also important to digital twins, a technology that aims to capture and simulate important properties and their interaction with the environment of physical objects. Implementing KG-based digital twins may greatly improve both treatment and research of mental disorders, as KGs could support more accurate capture of the wealth of information needed to perform simulations on digital twins (9).

In conclusion, while medical knowledge graphs have great potentials, several important issues need to be addressed in future work. Firstly, discovering new methods to enhance the interpretability of medical knowledge graphs should be a top priority. When dealing with the acquisition of missing unstructured information from limited databases, focusing on building a reliable information source is more crucial than simply completing it through technical means. Especially, in the field of neurological diseases where a professional and reliable database is lacking, this poses a significant challenge to the construction of professional KGs. Secondly, maintaining and updating data while reducing costs is also a problem that demands consideration at present. This requires exploring efficient strategies and technologies to ensure the accuracy and timeliness of medical knowledge graphs without incurring excessive expenses.
